# An estimation method for sensor faults based on observer in interconnected systems

**DOI:** 10.1371/journal.pone.0296848

**Published:** 2024-03-11

**Authors:** Yanxiu Sun, Hong Li

**Affiliations:** Basic Course Department, Shenyang Institute of Technology, Fushun, 113122, China; Institute of Theoretical and Applied Informatics Polish Academy of Sciences: Instytut Informatyki Teoretycznej i Stosowanej Polskiej Akademii Nauk, UKRAINE

## Abstract

In this research, a class of nonlinear interconnected systems with sensor faults were investigated and an estimation method was proposed for system sensor faults based on the theory of system state reconstruction. Considering sensor fault vectors in nonlinear interconnected systems, this method constructed a generalized nonlinear interconnected system, whose state was designed by augmenting the original system state and fault vectors, which provides a foundation for fault estimation of nonlinear interconnected systems. An augmented observer was developed by equivalent transformation of generalized interconnected system, so as to realize robust estimations of sensor faults in interconnected systems. This estimation method took into account the effect of external disturbance of the system on fault estimation and estimated the convergence speed of error system; the developed method also considered the convenience of solving the gain matrix of the augmented observer, which was beneficial to the realization of sensor fault estimation in interconnected system. The sensor estimation method proposed in the paper has the advantages of robustness in fault estimation,rapidity in error convergence, and convenience in solving the gain matrix. Finally, the state and sensor fault estimation errors of two interconnected systems can both approach 0 within 10 seconds, thus achieving the purpose of fault estimation. Two simulation experiments verified the effectiveness of the proposed fault estimation method and provided a reference for the fault estimation method of nonlinear interconnected systems with disturbance.

## 1. Introduction

For the past several decades, the scale and complexity of actual control systems have increased and research on interconnected systems has also has become increasingly popular among researchers around the world. Interconnected control systems are extensively applied in all aspects of society, such as production, power, transportation, multi-agent and other infrastructural systems [1,2]. Also, in case of sensor fault, actuator fault, and other system faults during the operation of interconnected systems, the subsystems or entire control system of interconnected systems do not function properly, which result in huge safety hazards. Therefore, the safety and stability of interconnected systems are of great importance, subsystem interfaces in interconnected systems make the estimation and diagnosis of fault in the entire system more difficult, and research on the estimation and diagnosis of faults in interconnected control systems has attracted widespread attention [3–5].

Observer-based fault detection and estimation methods have been wide-ranging used in fault detection technologies based on analytical redundancy [6–10]. The basic idea of these models is to reconstruct the system through system measurement output and use the difference between observer and system outputs as a residual to determine whether the system has malfunctioned. However, fault detection only qualitatively determines whether the system has malfunctioned, being unable to provide a deeper understanding of the fault. Fault reconstruction can directly display fault change process and diagnose deeper fault information.

Currently, certain results have been obtained from research on the stability and control issues of interconnected systems [11–21], and references [16–21] focus on the control problems of various interconnected systems such as power, and explore the decentralized control, robust control, optimal control, active control, and adaptive backstepping control problems of interconnected systems. Today, fault estimation and diagnosis methods for interconnected and complex systems are hot research topics. [22] proposed meta aircraft model through interconnected system models and designed centralized and distributed fault-tolerant control laws to ensure that the fault system could move from any initial state to the origin, making the fault system stable and providing conditions for the reconfiguration of the system. [23] applied adaptive estimation methods to estimate sensor fault for a class of uncertain discrete interconnected systems and realized fault estimation by designing an augmented observer due to the relaxed prerequisites of interval observers for systems and disturbances. For a generalized interconnected system, [24] proposed a design method for interval state observers. [25] studied fault estimation and fault-tolerant control problems in interconnected systems. [26] developed an estimation method for actuator faults in interconnected systems on the basis of distributed augmented observers and at the same time suppressed the influence of external disturbances in interconnected systems on actuator fault estimation. [27] investigated fault estimation of sensors in a certain control system and proposed a sliding mode observer with adaptive regulation law to detect and reconstruct sensor faults. [28] proposed an estimation method for robust state and fault based on neural networks for satellite attitude control systems with actuator and sensor faults. [29,30] have respectively studied the fault-tolerant control problem in large-scale interconnected power systems and propulsion systems for unmanned ships. [31,32] conducted research on multiple-input multiple-output (MIMO) antenna system. Sensors play an important role in interconnected systems and complex large-scale systems and the research on control methods, fault estimation, and diagnosis has important application significance.

Based on the above discussion, in this research, a sensor fault estimation method was proposed for a class of nonlinear interconnected systems with disturbances. By developing a nonlinear interconnected form of augmented observer, accurate estimations of sensor faults in each subsystem were achieved. Considering external disturbances, performance indicators were proposed to alleviate the effect of disturbances on fault estimation. Using MATLAB software, the gain matrix of the augmented observer could be easily solved. Finally, fault estimation in interconnected systems was realized and the feasibility of this method was verified by numerical simulation.

In summary, the contributions of this research were as follows:

- Considering sensor fault vector in nonlinear interconnected systems, an augmented vector was developed based on system state and fault and a generalized nonlinear interconnected system was proposed. The developed generalized interconnected system was transformed into normal form by equivalent transformation and an augmented observer was designed to ensure the feasibility of sensor fault estimation in interconnected systems.
- Sufficient conditions for the existence of augmented observers were given in two cases. Considering system nonlinearity and error system convergence speed, the conditions that the observer gain matrix needed to meet were given in the form of linear matrix inequality, which was convenient for solving the gain matrix. Considering the external disturbance characteristics of the system, the influence of external disturbance on observer estimation error could be effectively weakened by introducing performance indicators.
- In this paper, the fault estimation of a class of nonlinear interconnected systems was investigated and an effective fault estimation method was proposed. Also, two numerical examples were adopted to verify the effectiveness and feasibility of the proposed method, which provided a reference for fault estimation methods in interconnected systems with singularity, uncertainty and time delay.

The remaining of the paper is organized as follows: Section 2 describes the structure of nonlinear interconnected systems and provides conditions for fault estimation by introducing augmented vectors. In section 3, an observer design method was proposed based on equivalent transformation for state estimation in generalized interconnected systems. Section 4 introduces sufficient conditions for the existence of augmented state observer in the two cases. In observer design, both external disturbance and convergence rate of estimation error are considered. In section 5, two numerical examples are provided to simulate the fault estimation method proposed in this research and the effectiveness and feasibility of the proposed method are verified. Finally, section 6 summarizes the developed methods and puts forward future research path.

## 2. Problem statement and preliminaries

An interconnected system with N nonlinear subsystems was assumed as follows:

$$\{\begin{array}{l} {\dot{x}}_{i}(t)=A_{i}x_{i}(t)+\Phi_{i}(x_{i}(t),t)+B_{i}u_{i}(t)+D_{i}\xi_{i}(t)+\sum_{\begin{array}{l} j=1\\ j\neq i \end{array}}^{N}H_{ij}x_{j}(t)\\ y_{i}(t)=C_{i}x_{i}(t)+G_{i}f_{i}(t),i=1,2,\cdots ,N. \end{array}$$ (1)

where $x_{i}(t)\in R^{n}$, $u_{i}(t)\in R^{m}$, $y_{i}(t)\in R^{p}$, and $\xi_{i}(t)\in R^{l}$ are state vector, control input, controlled output and disturbance of the system, respectively, *f*_*i*_(*t*)∈R^*q*^ is sensor fault; *A*_*i*_, *B*_*i*_, *C*_*i*_, *D*_*i*_, *G*_*i*_ are known matrices with appropriate dimensions, *G*_*i*_ is full rank matrix for columns, *H*_*ij*_ is direct interconnection matrix between the *i*-th and *j*-th subsystems, and $\Phi_{i}(x_{i}(t),t)$ is the nonlinear term satisfying Lipschitz condition.

**Assumption 1.** The nonlinear term $\Phi_{i}(x_{i}(t),t)$ in the system satisfied Lipschitz condition as:

$$\|\Phi_{i}(x_{i}(t),t)-\Phi_{i}({\hat{x}}_{i}(t),t)\|\leq \gamma_{i}\|x_{i}-{\hat{x}}_{i}\|.$$ (2)

where *γ*_*i*_>0 is a real number.

**Assumption 2.** The column rank of matrix *G*_*i*_ was full.

**Lemma 1**[33] *ε* was considered as a scalar and *ε*>0, therefore:

$$2x^{T}y\leq \frac{1}{\varepsilon }x^{T}x+\varepsilon y^{T}y,\;x,y\in R^{n}.$$ (3)

**Lemma 2** (Schur complement lemma) [34]

The following conditions were equivalent for symmetric matrices $S=(\begin{array}{cc} S_{11} & S_{12}\\ S_{12}^{T} & S_{22} \end{array})$:

1. *S*<0;
2. $S_{11}<0,S_{22}-S_{12}^{T}S_{11}^{-1}S_{12}<0$;
3. $S_{22}<0,S_{11}-S_{12}S_{22}^{-1}S_{12}^{T}<0$.

This research focused on sensor fault estimation in interconnected system (1) and expanded state and fault vectors to obtain the new vector ${\bar{x}}_{i}=[\begin{array}{c} x_{i}(t)\\ f_{i}(t) \end{array}]$ giving the following equivalent system:

$$\{\begin{array}{l} E{\dot{\bar{x}}}_{i}(t)={\bar{A}}_{i}{\bar{x}}_{i}(t)+\Phi (E\bar{x}(t),t)+B_{i}u_{i}(t)+D_{i}\xi_{i}(t)+\sum_{\begin{array}{l} j=1\\ j\neq i \end{array}}^{N}H_{ij}E{\bar{x}}_{j}(t)\\ y_{i}(t)={\bar{C}}_{i}{\bar{x}}_{i}(t),\;i=1,2,\cdots ,N. \end{array}$$ (4)

where $E=[I_{n}\;0]$, ${\bar{A}}_{i}=[A_{i}\;0]$, ${\bar{C}}_{i}=[C_{i}\;G_{i}]$ and matrix 0 is proper dimension zero matrix in augmented matrix.

**Remark 1.** For a class of nonlinear interconnected systems, a distributed fault estimation observer was designed according to information between interconnected subsystems to perform online real-time fault estimation in each subsystem.

**Remark 2.** Nonlinear interconnected systems contained disturbance terms. Considering the effect of system disturbance terms on fault estimation, this research adopted the idea of robust estimation. By setting performance indicators, disturbance impact on fault estimation was minimized as much as possible.

## 3.Design of augmented observer for nonlinear interconnected systems

If matrix *G*_*i*_ was a column full rank matrix, it was known that $\left(\begin{array}{c} E\\ {\bar{C}}_{i} \end{array}\right)$ was also a column full rank Matrix.

Let

$$(S_{i}T_{i})={\left[{\left(\begin{array}{c} E\\ {\bar{C}}_{i} \end{array}\right)}^{T}(\begin{array}{c} E\\ {\bar{C}}_{i} \end{array})\right]}^{-1}{\left(\begin{array}{c} E\\ {\bar{C}}_{i} \end{array}\right)}^{T}.$$ (5)

so

$$S_{i}E+T_{i}{\bar{C}}_{i}=I_{n+q},\;i=1,2,\cdots ,N.$$ (6)

and

$${\dot{\bar{x}}}_{i}(t)=S_{i}{\bar{A}}_{i}{\bar{x}}_{i}(t)+S_{i}\Phi_{i}(E{\bar{x}}_{i}(t),t)+S_{i}B_{i}u_{i}(t)+S_{i}D_{i}\xi_{i}(t)+S_{i}\sum_{\begin{array}{l} j=1\\ j\neq i \end{array}}^{N}H_{ij}E{\bar{x}}_{j}(t)+T_{i}{\dot{y}}_{i}(t)$$ (7)

In order to estimate sensor faults in the interconnected system (1), this paper designed the following observer to achieve robust fault vector estimation:

$$\{\begin{array}{l} {\dot{\eta }}_{i}(t)=S_{i}{\bar{A}}_{i}\eta_{i}(t)+S_{i}{\bar{A}}_{i}T_{i}y_{i}(t)+S_{i}\Phi_{i}(E{\hat{\bar{x}}}_{i}(t),t)+S_{i}B_{i}u_{i}(t)+S_{i}\sum_{\begin{array}{l} j=1\\ j\neq i \end{array}}^{N}H_{ij}E{\hat{\bar{x}}}_{j}(t)+L_{i}(y_{i}(t)-{\hat{y}}_{i}(t)),\\ {\hat{\bar{x}}}_{i}(t)=\eta_{i}(t)+T_{i}y_{i}(t),\\ {\hat{y}}_{i}(t)={\bar{C}}_{i}{\hat{\bar{x}}}_{i}(t).\\ i=1,2,\cdots ,N. \end{array}$$ (8)

where *η*_*i*_(*t*), ${\hat{y}}_{i}(t)$ and *L*_*i*_ are observer state, output vector estimation and gain matrix of the observer to be designed, respectively.

State and sensor fault estimation errors were defined as $e_{x_{i}}(t)=x_{i}(t)-{\hat{x}}_{i}(t)$ and $e_{i}(t)={\bar{x}}_{i}(t)-{\hat{\bar{x}}}_{i}(t)$. Then, the estimation error of augmented state was expressed as $e_{i}(t)=[\begin{array}{c} e_{x_{i}}(t)\\ e_{f_{i}}(t) \end{array}]$. For limit $\underset{t\to \infty }{\lim}e_{i}(t)=0$, one could get $\underset{t\to \infty }{\lim}e_{x_{i}}(t)=0,\underset{t\to \infty }{\lim}e_{f_{i}}(t)=0$.

The design of the above-mentioned augmented observer was able to simultaneously obtain estimates of state and sensor faults of the original interconnected system.

According to Eqs (3) and (4), the error dynamic system of the following subsystem 1 was obtained as:

$${\dot{e}}_{i}(t)={\dot{\bar{x}}}_{i}(t)-{\dot{\hat{\bar{x}}}}_{i}(t)$$

$$=S_{i}{\bar{A}}_{i}{\bar{x}}_{i}(t)+S_{i}\Phi_{i}(E{\bar{x}}_{i}(t),t)+S_{i}B_{i}u_{i}(t)+S_{i}D_{i}\xi_{i}(t)+S_{i}\sum_{\begin{array}{l} j=1\\ j\neq i \end{array}}^{N}H_{ij}E{\bar{x}}_{j}(t)+T_{i}{\dot{y}}_{i}(t)-{\dot{\eta }}_{i}(t)-T_{i}{\dot{y}}_{i}(t)$$

$$=S_{i}{\bar{A}}_{i}{\bar{x}}_{i}(t)+S_{i}\Phi_{i}(E{\bar{x}}_{i}(t),t)+S_{i}D_{i}\xi_{i}(t)+S_{i}\sum_{\begin{array}{l} j=1\\ j\neq i \end{array}}^{N}H_{ij}E{\bar{x}}_{j}(t)-S_{i}{\bar{A}}_{i}{\hat{\bar{x}}}_{i}(t)-S_{i}\Phi_{i}(E{\hat{\bar{x}}}_{i}(t),t)-$$

$$S_{i}\sum_{\begin{array}{l} j=1\\ j\neq i \end{array}}^{N}H_{ij}E{\hat{\bar{x}}}_{j}(t)-L_{i}(y_{i}(t)-{\bar{C}}_{i}{\hat{\bar{x}}}_{i}(t))$$

$$=(S_{i}{\bar{A}}_{i}-L_{i}{\bar{C}}_{i})e_{i}(t)+\Delta \Phi_{i}+S_{i}D_{i}\xi_{i}(t)+S_{i}\sum_{\begin{array}{l} j=1\\ j\neq i \end{array}}^{N}H_{ij}Ee_{j}(t)$$ (9)

where $\Delta \Phi_{i}=S_{i}(\Phi_{i}(E{\bar{x}}_{i}(t),t)-\Phi_{i}(E{\hat{\bar{x}}}_{i}(t),t))$.

The purpose of this work was to estimate sensor faults in the original interconnected system by designing an augmented observer (8).

## 4. Results

In this research, the designed augmented observer was analyzed and the existence conditions of the observer were introduced in the form of matrix inequality, so as to ensure that the gain matrix of the augmented observer could be calculated conveniently by using MATLAB software and thus, sensor fault in interconnected system could be estimated.

Here, the existence conditions of observers have been discussed for two cases and in order to facilitate the system fault estimation, the sufficient conditions for the existence of the observer are given in the form of matrix inequalities in the design of the augmented observer.

### 4.1 Existence conditions of augmented observer when the system did not contain nonlinear and disturbance terms

**Theorem 1.** *If there existed a symmetric positive definite matrix P*_*i*_ *and a gain matrix L*_*i*_ *in observer (8)*, *the following conditions were met*:

$$(\begin{array}{cccc} {\tilde{M}}_{11} & {\tilde{M}}_{12} & \cdots & {\tilde{M}}_{1N}\\ {}* & {\tilde{M}}_{22} & \cdots & {\tilde{M}}_{2N}\\ {}* & * & \ddots & \vdots \\ {}* & * & * & {\tilde{M}}_{NN} \end{array})<0$$ (10)

Where

$${\tilde{M}}_{ii}={{\bar{A}}_{i}}^{T}{S_{i}}^{T}P_{i}-{{\bar{C}}_{i}}^{T}{Y_{i}}^{T}+P_{i}S_{i}{\bar{A}}_{i}-Y_{i}{\bar{C}}_{i},$$

$${\tilde{M}}_{ij}=P_{i}S_{i}H_{ij}E+E^{T}H_{ji}^{T}{S_{j}}^{T}P_{j},$$

$$Y_{i}=P_{i}L_{i},i\leq j;i=1,2,\cdots ,N;j=1,2,\cdots ,N.$$

Then, the estimation errors of system state and sensor fault asymptotically approached zero at the same time and the augmented observe (8) realized robust estimation of sensor fault in the linear interconnected system without disturbance term.

**Proof.** Lyapunov–Krasovskii functional was considered as $V=\sum_{i=1}^{N}{e_{i}}^{T}P_{i}e_{i}$.

Then,

$$\begin{array}{l} \dot{V}=\sum_{i=1}^{N}\left({{\dot{e}}_{i}}^{T}P_{i}e_{i}+{e_{i}}^{T}P_{i}{\dot{e}}_{i}\right)\\ \;=\sum_{i=1}^{N}\{[{e_{i}}^{T}{\left(S_{i}{\bar{A}}_{i}-L_{i}{\bar{C}}_{i}\right)}^{T}+(S_{i}\sum_{\begin{array}{l} j=1\\ j\neq i \end{array}}^{N}H_{ij}Ee_{j}{)}^{T}]P_{i}e_{i}+{e_{i}}^{T}P_{i}[(S_{i}{\bar{A}}_{i}-L_{i}{\bar{C}}_{i})e_{i}+S_{i}\sum_{\begin{array}{l} j=1\\ j\neq i \end{array}}^{N}H_{ij}Ee_{j}]\}\\ \;=\sum_{i=1}^{N}\{{e_{i}}^{T}[{\left(S_{i}{\bar{A}}_{i}-L_{i}{\bar{C}}_{i}\right)}^{T}P_{i}+P_{i}(S_{i}{\bar{A}}_{i}-L_{i}{\bar{C}}_{i})]e_{i}+2{e_{i}}^{T}P_{i}S_{i}\sum_{\begin{array}{l} j=1\\ j\neq i \end{array}}^{N}H_{ij}Ee_{j}) \end{array}$$

$$={\left(\begin{array}{c} e_{1}\\ e_{2}\\ \vdots \\ e_{N} \end{array}\right)}^{T}(\begin{array}{cccc} M_{11} & M_{12} & \cdots & M_{1N}\\ {}* & M_{22} & \cdots & M_{2N}\\ {}* & * & \ddots & \vdots \\ {}* & * & * & M_{NN} \end{array})(\begin{array}{c} e_{1}\\ e_{2}\\ \vdots \\ e_{N} \end{array}).$$ (11)

where

$$M_{ii}={\left(S_{i}{\bar{A}}_{i}-L_{i}{\bar{C}}_{i}\right)}^{T}P_{i}+P_{i}(S_{i}{\bar{A}}_{i}-L_{i}{\bar{C}}_{i}),$$

$$M_{ij}=P_{i}S_{i}H_{ij}E+E^{T}H_{ji}^{T}{S_{j}}^{T}P_{j},$$

$$Y_{i}=P_{i}L_{i},\;i\leq j;\;i=1,2,\cdots ,N;j=1,2,\cdots ,N.$$

According to Lemma 2 Matrix inequality $(\begin{array}{cccc} M_{11} & M_{12} & \cdots & M_{1N}\\ {}* & M_{22} & \cdots & M_{2N}\\ {}* & * & \ddots & \vdots \\ {}* & * & * & M_{NN} \end{array})<0$ equivalent to inequality (6), proof was finished.

**Remark 3.** The above theorem gave the existence conditions of augmented observer when the system had no external disturbance and the relationship was linear. This type of system was relatively simple and in practical applications, the system had more complexity such as concrete nonlinearity and external disturbance. At the same time, estimation error convergence speed was also an index to be considered when estimating system faults. In the next section, conditions such as nonlinear terms and external disturbances in the system will be taken into account, along with a discussion of the speed of convergence of the fault error estimates.

### 4.2 Existence conditions of augmented observers when the interconnected system was nonlinear and contained external disturbance

**Theorem 2.** *If there existed a symmetric positive definite matrix P*_*i*_, *Q*_*i*_ *and a gain matrix L*_*i*_ *in observer (8)*, *the following conditions were met*:

$$(\begin{array}{cccc} {\tilde{\bar{M}}}_{11} & {\tilde{\bar{M}}}_{12} & \cdots & {\tilde{\bar{M}}}_{1N}\\ {}* & {\tilde{\bar{M}}}_{22} & \cdots & {\tilde{\bar{M}}}_{2N}\\ {}* & * & \ddots & \vdots \\ {}* & * & * & {\tilde{\bar{M}}}_{NN} \end{array})<0.$$ (12)

Where

$${\tilde{\bar{M}}}_{ii}=(\begin{array}{ccc} {{\bar{A}}_{i}}^{T}{S_{i}}^{T}P_{i}-{{\bar{C}}_{i}}^{T}{Y_{i}}^{T}+P_{i}S_{i}{\bar{A}}_{i}-Y_{i}{\bar{C}}_{i}+{\beta_{i}}^{2}I+I^{T}I+Q_{i} & P_{i} & P_{i}S_{i}D_{i}\\ {}* & -I & 0\\ {}* & * & -\alpha^{2}I \end{array}),$$

$${\tilde{\bar{M}}}_{ij}=(\begin{array}{ccc} P_{i}S_{i}H_{ij}E+E^{T}H_{ji}^{T}{S_{j}}^{T}P_{j} & 0 & 0\\ {}* & 0 & 0\\ {}* & * & 0 \end{array}),$$

$$Y_{i}=P_{i}L_{i},i\leq j;i=1,2,\cdots ,N;j=1,2,\cdots ,N.$$

*Then, the augmented state observer (8) could make the augmented state estimation error converge exponentially with stability margin by $\frac{\delta_{0}}{2}$, and realize robust estimation of sensor fault in the nonlinear interconnected system (1). Among them,*

$$\delta_{0}=\frac{\lambda_{\min}(Q)}{\lambda_{\max}(P)},$$

$$\lambda_{\min}(Q)=\min\{\lambda_{\min}(Q_{1}),\lambda_{\min}(Q_{2}),\cdots ,\lambda_{\min}(Q_{N})\},$$

$$\lambda_{\max}(P)=\max\{\lambda_{\min}(P_{1}),\lambda_{\min}(P_{2}),\cdots ,\lambda_{\min}(P_{N})\}.$$

*where λ*_min_(*X*), *λ*_max_(*X*), *represent minimum and maximum eigenvalues of matrix X*, *respectively*.

**Proof.** Lyapunov–Krasovskii functional was considered as $V=\sum_{i=1}^{N}{e_{i}}^{T}P_{i}e_{i}$.

Then,

$$\dot{V}=\sum_{i=1}^{N}({{\dot{e}}_{i}}^{T}P_{i}e_{i}+{e_{i}}^{T}P_{i}{\dot{e}}_{i})$$

$$=\sum_{i=1}^{N}\{[{e_{i}}^{T}{\left(S_{i}{\bar{A}}_{i}-L_{i}{\bar{C}}_{i}\right)}^{T}+\Delta {\Phi_{i}}^{T}+{\left(S_{i}D_{i}\xi_{i}\right)}^{T}+(S_{i}\sum_{\begin{array}{l} j=1\\ j\neq i \end{array}}^{N}H_{ij}Ee_{j}{)}^{T}]P_{i}e_{i}+{e_{i}}^{T}P_{i}[(S_{i}{\bar{A}}_{i}-L_{i}{\bar{C}}_{i})e_{i}+$$

$$\Delta \Phi_{i}+S_{i}D_{i}\xi_{i}+S_{i}\sum_{\begin{array}{l} j=1\\ j\neq i \end{array}}^{N}H_{ij}Ee_{j}]\}$$

$$=\sum_{i=1}^{N}\{{e_{i}}^{T}[{\left(S_{i}{\bar{A}}_{i}-L_{i}{\bar{C}}_{i}\right)}^{T}P_{i}+P_{i}(S_{i}{\bar{A}}_{i}-L_{i}{\bar{C}}_{i})]e_{i}+2{e_{i}}^{T}P_{i}\Delta \Phi_{i}+2{e_{i}}^{T}P_{i}S_{i}D_{i}\xi_{i}+2{e_{i}}^{T}P_{i}S_{i}\sum_{\begin{array}{l} j=1\\ j\neq i \end{array}}^{N}H_{ij}Ee_{j}\}$$ (13)

According to Hypothesis 1 and Lemma 1, the following inequality was obtained:

$$2{e_{i}}^{T}P_{i}\Delta \Phi_{i}\leq {e_{i}}^{T}P_{i}P_{i}e_{i}+{\beta_{i}}^{2}{e_{i}}^{T}e_{i}.$$ (14)

where *β*_*i*_ = *γ*_*i*_‖*S*_*i*_‖

Thus,

$$\dot{V}\leq \sum_{i=1}^{N}\{{e_{i}}^{T}[(S_{i}{\bar{A}}_{i}-L_{i}{\bar{C}}_{i}{)}^{T}P_{i}+P_{i}(S_{i}{\bar{A}}_{i}-L_{i}{\bar{C}}_{i})+{P_{i}}^{T}P_{i}+{\beta_{i}}^{2}I]e_{i}-2{e_{i}}^{T}P_{i}S_{i}D_{i}\xi_{i}+2{e_{i}}^{T}P_{i}S_{i}\sum_{\begin{array}{l} j=1\\ j\neq i \end{array}}^{N}H_{ij}Ee_{j}\}.$$ (15)

When $\dot{V}<0$, the asymptotic convergence of state estimation error was guaranteed. In order to more effectively improve error system convergence speed, the following methods were adopted in this paper.

If there exist matrices $Q_{i}>0(i=1,2,\cdots ,N)$, $\dot{V}<-\sum_{i=1}^{N}{e_{i}}^{T}Q_{i}e_{i}$ was satisfied and the convergence speed of error system was accelerated.


$$\dot{V}<-\sum_{i=1}^{N}{e_{i}}^{T}Q_{i}e_{i}\leq -\lambda_{\min}(Q)\sum_{i=1}^{N}{\left\|e_{i}\right\|}^{2}$$ (16)

$$V=\sum_{i=1}^{N}{e_{i}}^{T}P_{i}e_{i}\geq \lambda_{\min}(P)\sum_{i=1}^{N}{\left\|e_{i}\right\|}^{2}$$ (17)

Where

$$\lambda_{\max}(P)=\max\{\lambda_{\min}(P_{1}),\lambda_{\min}(P_{2}),\cdots ,\lambda_{\min}(P_{N})\}$$

$$V=\sum_{i=1}^{N}{e_{i}}^{T}P_{i}e_{i}\leq \lambda_{\max}(P)\sum_{i=1}^{N}{\left\|e_{i}\right\|}^{2}$$ (18)

Where

$$\lambda_{\max}(P)=\max\{\lambda_{\min}(P_{1}),\lambda_{\min}(P_{2}),\cdots ,\lambda_{\min}(P_{N})\}$$

According to Eqs (17) and (18):

$$\sum_{i=1}^{N}{\left\|e_{i}\right\|}^{2}\leq \frac{V}{\lambda_{\min}(P)},\;\sum_{i=1}^{N}{\left\|e_{i}\right\|}^{2}\geq \frac{V}{\lambda_{\max}(P)}$$ (19)

And according to Eqs (16) and (19):

$$\dot{V}<-\lambda_{\min}(Q)\sum_{i=1}^{N}{\left\|e_{i}\right\|}^{2}\leq -\lambda_{\min}(Q)\frac{V}{\lambda_{\min}(P)}$$

$$=-\frac{\lambda_{\min}(Q)}{\lambda_{\min}(P)}V=-\delta_{0}V,(\delta_{0}=\frac{\lambda_{\min}(Q)}{\lambda_{\min}(P)})$$ (20)

According to (20):

$$V\leq e^{-\delta_{0}t}V(0)$$ (21)

And according to Eqs (19) and (21):

$$\sum_{i=1}^{N}{\left\|e_{i}\right\|}^{2}\leq \frac{V}{\lambda_{\min}(P)}\leq \frac{e^{-\delta_{0}t}V(0)}{\lambda_{\min}(P)}=\frac{V(0)}{\lambda_{\min}(P)}e^{-\delta_{0}t}$$ (22)

To sum up, if there were matrix *P*_*i*_, *Q*_*i*_, *L*_*i*_ and Eq (12) was satisfied, then $\underset{t\to \infty }{\lim}\sum_{i=1}^{N}{\left\|e_{i}\right\|}^{2}=0$ and convergence speed was greater than $e^{-\delta_{0}t}$.

**Remark 4.** When designing an augmented observe, one could choose *P*_*i*_ and *Q*_*i*_ correlation; for example, *Q*_*i*_ = *αP*_*i*_ to ensure the convergence speed of the obtained observer.

In order to decrease the effect of interference on sensor faults, it was assumed that

$$\tilde{V}=\dot{V}+\sum_{i=1}^{N}{e_{i}}^{T}Q_{i}e_{i}+\sum_{i=1}^{N}\left({e_{i}}^{T}e_{i}-\alpha^{2}{\xi_{i}}^{T}\xi_{i}\right).$$ (23)

The following inequalities are always true:

$$\tilde{V}<0,$$

$$\int_{0}^{+\infty }\left[\dot{V}+\sum_{i=1}^{N}{e_{i}}^{T}Q_{i}e_{i}+\sum_{i=1}^{N}\left({e_{i}}^{T}e_{i}-\alpha^{2}{\xi_{i}}^{T}\xi_{i}\right)\right]dt<0,$$

$$\int_{0}^{+\infty }\sum_{i=1}^{N}{e_{i}}^{T}e_{i}dt-\alpha^{2}\int_{0}^{\infty }\sum_{i=1}^{N}{\xi_{i}}^{T}\xi_{i}dt<0,$$ (24)

$$\|e_{f}\|<\|e\|,$$ (25)

‖e‖<α‖ξ‖, (26)

Where $e_{f}={\left[\begin{array}{cccc} e_{f_{1}}^{T} & e_{f_{2}}^{T} & \cdots & e_{f_{N}}^{T} \end{array}\right]}^{T}$, $e={\left[\begin{array}{cccc} e_{1}^{T} & e_{2}^{T} & \cdots & e_{N}^{T} \end{array}\right]}^{T}$, $\xi ={\left[\begin{array}{cccc} \xi_{1}^{T} & \xi_{2}^{T} & \cdots & \xi_{N}^{T} \end{array}\right]}^{T}$.

According to Eqs (24), (25) and (26):

$$\|e_{f}\|<\alpha \|\xi \|.$$ (27)

Parameter *α* could be adjusted to reduce the influence of disturbance on fault estimation and thus, a robust sensor fault estimation in the subsystem could be obtained.

Let $X_{i}=[\begin{array}{c} e_{i}(t)\\ \xi_{i}(t) \end{array}]$, then

$$\tilde{V}=\dot{V}+\sum_{i=1}^{N}{e_{i}}^{T}Q_{i}e_{i}+\sum_{i=1}^{N}\left({e_{i}}^{T}e_{i}-\alpha^{2}{\xi_{i}}^{T}\xi_{i}\right)$$

$$\leq \sum_{i=1}^{N}\{{e_{i}}^{T}[{\left(S_{i}{\bar{A}}_{i}-L_{i}{\bar{C}}_{i}\right)}^{T}P_{i}+P_{i}(S_{i}{\bar{A}}_{i}-L_{i}{\bar{C}}_{i})+{P_{i}}^{T}P_{i}+{\beta_{i}}^{2}I+I^{T}I+Q_{i}]e_{i}-2{e_{i}}^{T}P_{i}S_{i}D_{i}\xi_{i}+2{e_{i}}^{T}P_{i}S_{i}\sum_{\begin{array}{l} j=1\\ j\neq i \end{array}}^{N}H_{ij}Ee_{j}-\alpha^{2}{\xi_{i}}^{T}\xi_{i}\}$$

$$={\left(\begin{array}{c} X_{1}\\ X_{2}\\ \vdots \\ X_{N} \end{array}\right)}^{T}(\begin{array}{cccc} {\bar{M}}_{11} & {\bar{M}}_{12} & \cdots & {\bar{M}}_{1N}\\ {}* & {\bar{M}}_{22} & \cdots & {\bar{M}}_{2N}\\ {}* & * & \ddots & \vdots \\ {}* & * & * & {\bar{M}}_{NN} \end{array})(\begin{array}{c} X_{1}\\ X_{2}\\ \vdots \\ X_{N} \end{array}).$$ (28)

Where

$${\bar{M}}_{ii}=(\begin{array}{cc} {\left(S_{i}{\bar{A}}_{i}-L_{i}{\bar{C}}_{i}\right)}^{T}P_{i}+P_{i}(S_{i}{\bar{A}}_{i}-L_{i}{\bar{C}}_{i})+{P_{i}}^{T}P_{i}+{\beta_{i}}^{2}I+I^{T}I+Q_{i} & P_{i}S_{i}D_{i}\\ {}* & -\alpha^{2}I \end{array}),$$

$${\bar{M}}_{ij}=(\begin{array}{cc} P_{i}S_{i}H_{ij}E+E^{T}H_{ji}^{T}{S_{j}}^{T}P_{j} & 0\\ {}* & 0 \end{array}),$$

i≤j;i=1,2,⋯,N;j=1,2,⋯,N.

According to Lemma 2 Matrix inequality $(\begin{array}{cccc} {\bar{M}}_{11} & {\bar{M}}_{12} & \cdots & {\bar{M}}_{1N}\\ {}* & {\bar{M}}_{22} & \cdots & {\bar{M}}_{2N}\\ {}* & * & \ddots & \vdots \\ {}* & * & * & {\bar{M}}_{NN} \end{array})<0$ equivalent to inequality (12), proof was finished.

**Remark 5.** When designing interconnected system observers, it is difficult to deal with interconnection term, and at the same time, it is necessary to take into account the interference term of the system. This theorem determined the conditions for the existence of augmented observer in the form of linear matrix inequality, which was convenient for solving the gain matrix. Ultimately, robust estimation of sensor faults could be achieved in each subsystem by the augmented observer.

### 4.3 Sensor fault estimation of nonlinear interconnected systems

Through theorem proof, the estimated value ${\hat{\bar{x}}}_{i}(t)$ of the state vector ${\bar{x}}_{i}(t)$ was obtained by the designed observer. Obviously, the relationship ${\hat{f}}_{i}(t)=[0\;I_{q}]{\hat{\bar{x}}}_{i}$ was obtained based on the augmented state vector.

The designed gain matrix was solved by inequality (6) to design the augmented observer of interconnected system. Finally, the sensor fault estimation of interconnected system (1) was realized by relational expression ${\hat{f}}_{i}(t)=[\begin{array}{cc} 0 & I_{q} \end{array}]{\hat{\bar{x}}}_{i}$.

## 5. Illustrative examples

### 5.1 Simulation verification of interconnected system without nonlinear term and disturbance term

Interconnected system 1: Referring to the example in reference [9], the following two subsystems with sensor faults were considered.

The parameter matrix of subsystem 1 was considered as:

$$A_{1}=(\begin{array}{cc} -1 & 1\\ 0 & 0 \end{array}),\;B_{1}=(\begin{array}{cc} 1 & 0\\ 0 & 1 \end{array}),\;C_{1}=(\begin{array}{cc} 1 & 0\\ 0 & 1 \end{array}),$$

$$H_{12}=(\begin{array}{cc} -1 & 0\\ 1 & 0 \end{array}),\;G_{1}=(\begin{array}{c} 0.1\\ 0 \end{array}).$$

The parameter matrix of subsystem 2 was assumed as:

$$A_{2}=(\begin{array}{cc} -1 & 1\\ -4 & -3 \end{array}),\;B_{2}=(\begin{array}{cc} 1 & 0\\ 0 & 1 \end{array}),\;C_{2}=(\begin{array}{cc} 1 & 0\\ 0 & 1 \end{array}),$$

$$H_{21}=(\begin{array}{cc} -1 & 0\\ -1 & -2 \end{array}),\;G_{2}=(\begin{array}{c} 0.1\\ 0 \end{array}).$$

According to theorem 1 in part 4, the gain matrix of the augmented observe of two subsystems was calculated via

Matlab LMI Control Toolbox.


$$L_{1}=(\begin{array}{cc} -36.9392 & 31.5205\\ -14.2585 & -40.1072\\ -7.5525 & 10.7837 \end{array}),\;L_{2}=(\begin{array}{cc} -21.1207 & 10.7632\\ 7.8242 & -3.5047\\ -4.0123 & 2.9451 \end{array}).$$

When the initial values of the generalized state estimation errors of the two subsystems in the interconnected system were $\left[\begin{array}{ccc} -0.5 & 0.5 & 0.2 \end{array}\right]$ and $\left[\begin{array}{ccc} -0.4 & 0.3 & 0.2 \end{array}\right]$, the following state response curves were obtained.

**Fig 1:**
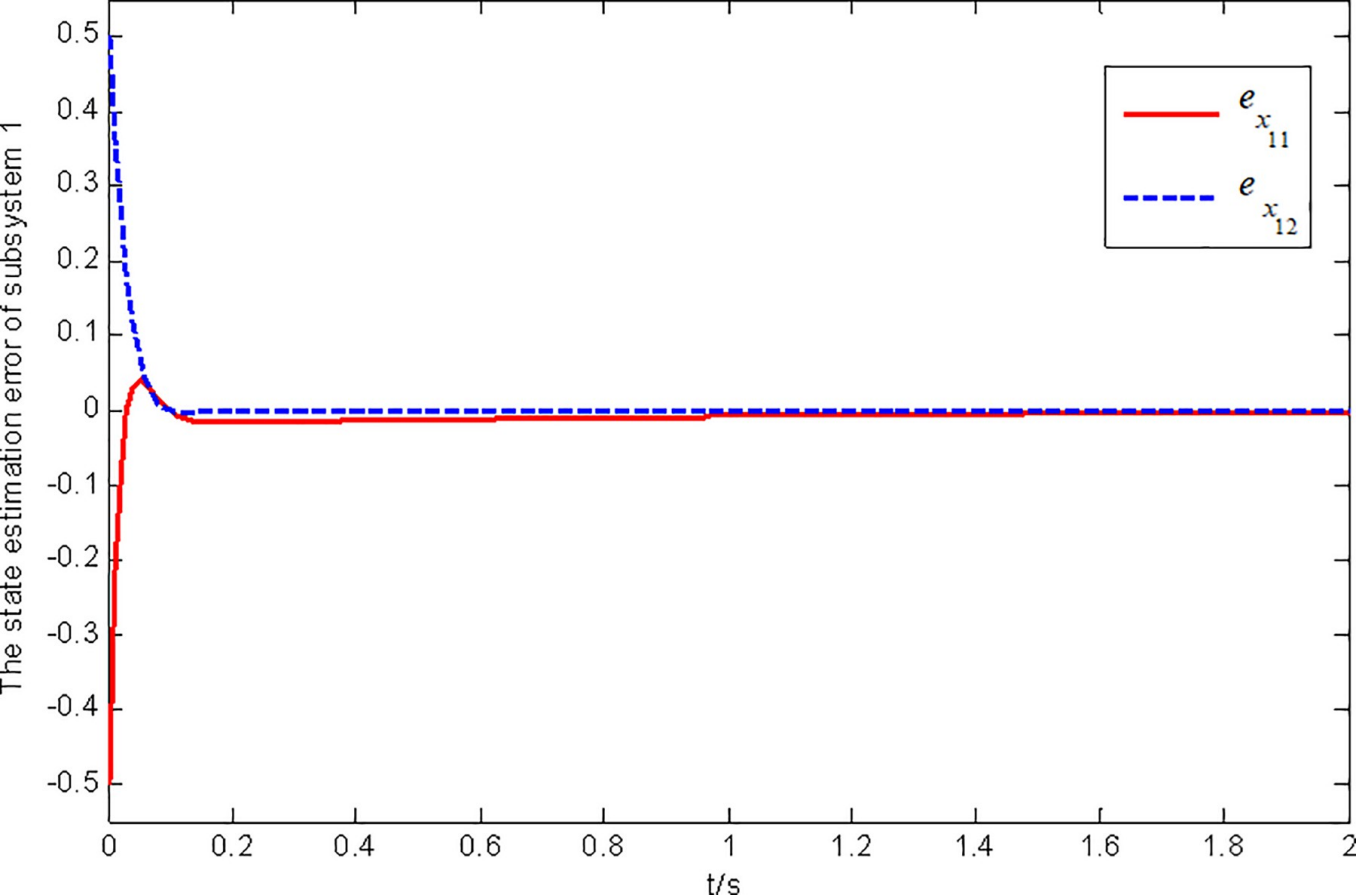
State estimation error response of subsystem 1 in interconnected system 1.

**Fig 2:**
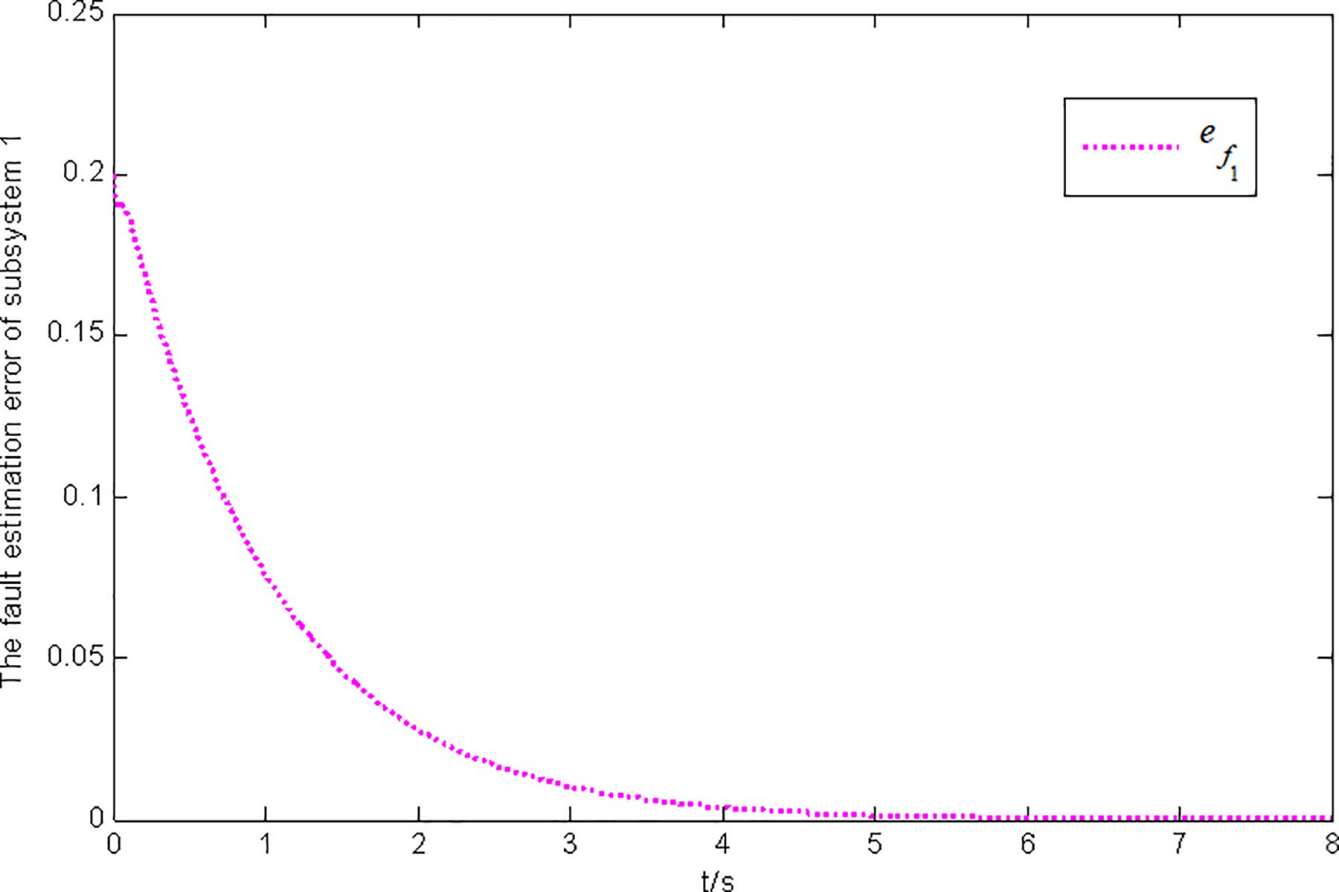
Fault estimation error response of subsystem 1 in interconnected system 1.

Figs 1 and 2 show the state and fault estimation error response curves of subsystem 1 in interconnected system 1.

**Fig 3:**
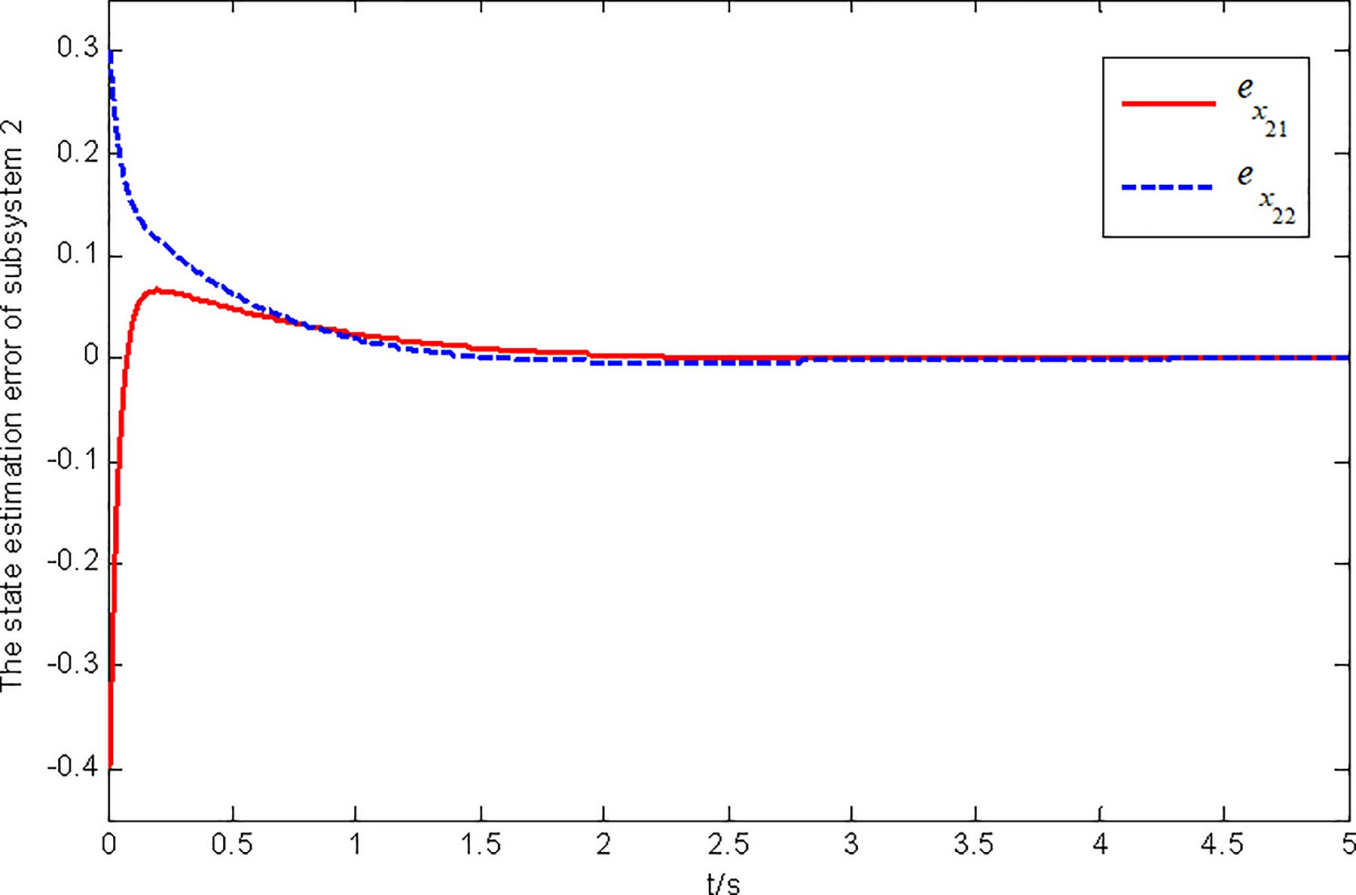
State estimation error response of subsystem 2 in interconnected system 1.

**Fig 4:**
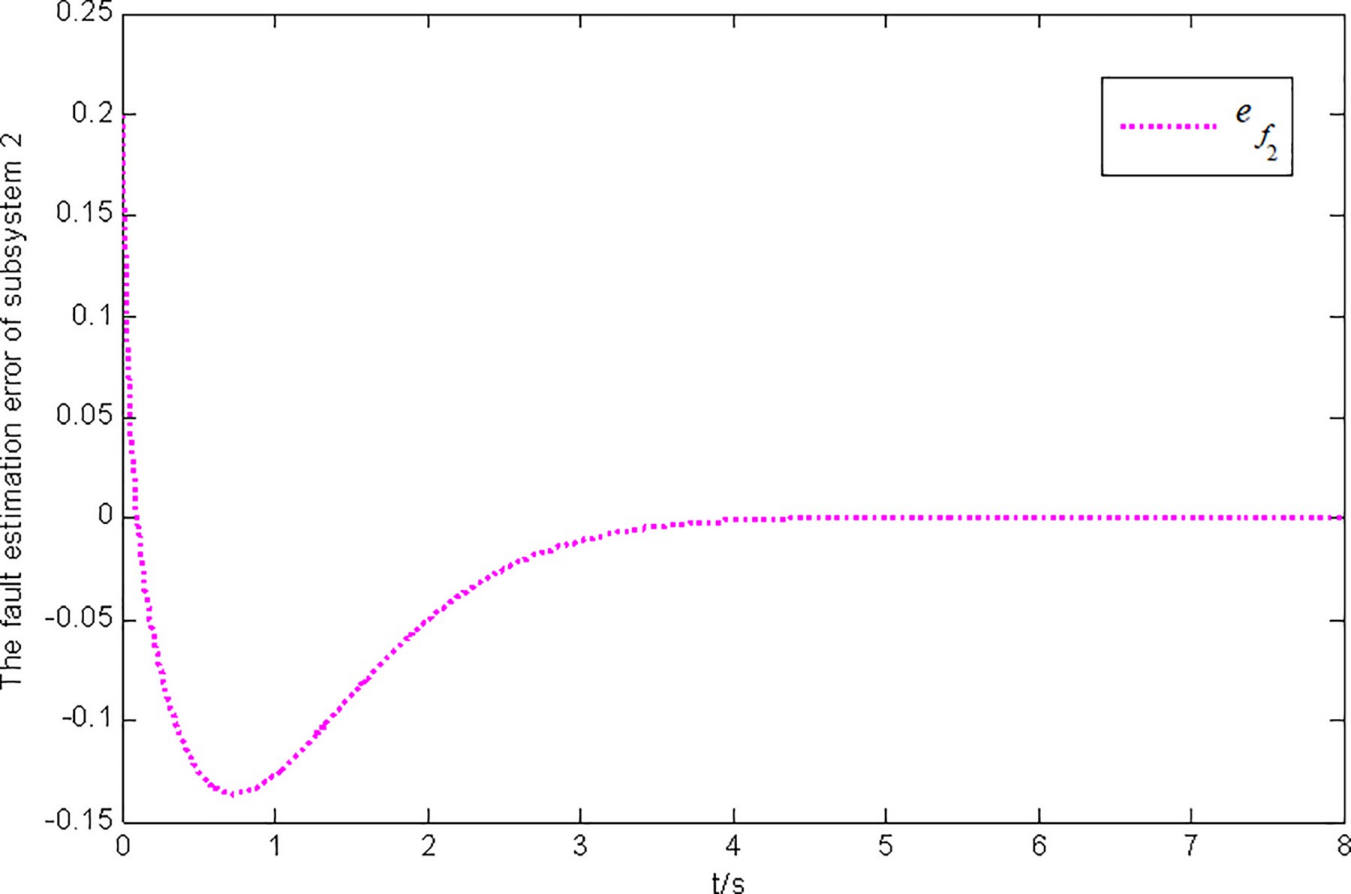
Fault estimation error response of subsystem 2 in interconnected system 1.

Figs 3 and 4 present the state and fault estimation error response curves of subsystem 2 in interconnected system 1.

### 5.2 Simulation verification of interconnected system with nonlinear and disturbance terms

Interconnected system 2: Referring to the example in reference [10], the following two subsystems with sensor faults were considered.

The parameter matrix of subsystem 1 was:

$$A_{1}=(\begin{array}{ccc} -3 & 0 & 1\\ 1 & -1 & 2\\ 1 & 0 & -6 \end{array}),\;B_{1}=(\begin{array}{ll} 1 & 0\\ 0 & 1\\ 0 & 0 \end{array}),\;C_{1}=(\begin{array}{lll} 1 & 0 & 0\\ 0 & 1 & 0 \end{array}),$$

$$D_{1}=(\begin{array}{c} 0.01\\ 0.01\\ 0.01 \end{array}),\;\Phi_{1}(x_{1}(t),t)=(\begin{array}{c} 0\\ 0\\ 0.5\sin x_{12}(t) \end{array}),$$

$$H_{12}=(\begin{array}{ccc} 0 & 0.1 & 0\\ 0 & 0 & 0.1\\ 0.1 & 0 & 0 \end{array}),\;G_{1}=(\begin{array}{c} 1\\ 0 \end{array}).$$

The parameter matrix of subsystem 2 was:

$$A_{2}=(\begin{array}{ccc} -2 & 0 & 1\\ 0 & -5 & 2\\ 2 & 1 & -6 \end{array}),\;B_{2}=(\begin{array}{ll} 1 & 0\\ 0 & 1\\ 0 & 0 \end{array}),\;C_{2}=(\begin{array}{lll} 1 & 0 & 0\\ 0 & 1 & 0 \end{array}),$$

$$D_{2}=(\begin{array}{c} 0.01\\ 0.01\\ 0.01 \end{array}),\;\Phi_{2}(x_{2}(t),t)=(\begin{array}{c} 0\\ 0\\ 0.4\sin x_{23}(t) \end{array}),$$

$$H_{21}=(\begin{array}{ccc} 0 & 0.1 & 0\\ 0 & 0.1 & 0\\ 0 & 0 & 0.1 \end{array}),\;G_{1}=(\begin{array}{c} 0\\ 1 \end{array}).$$

In this example, it was assumed that *Q*_*i*_ = *P*_*i*_ and the linear matrix inequality (12) was solved. According to *Y*_*i*_ = *P*_*i*_*L*_*i*_, the gain matrix of augmented observe of two subsystems was calculated by MATLAB.

When the initial value of the state estimation error in interconnected system 2 was $\left[\begin{array}{cccc} -0.4 & -0.2 & 0.3 & 0.3 \end{array}\right]$, the following response curves for the state and fault estimation errors in the system were given by solving the gain matrices of the two subsystems.

(1) Gain matrix of augmented observe in the first subsystem was assumed as:

$$L_{1}=(\begin{array}{cc} -0.0137 & -5.2483\\ 4.9902 & -1.3187\\ 0.0685 & -0.2411\\ -1.4187 & -5.0045 \end{array}).$$

**Fig 5:**
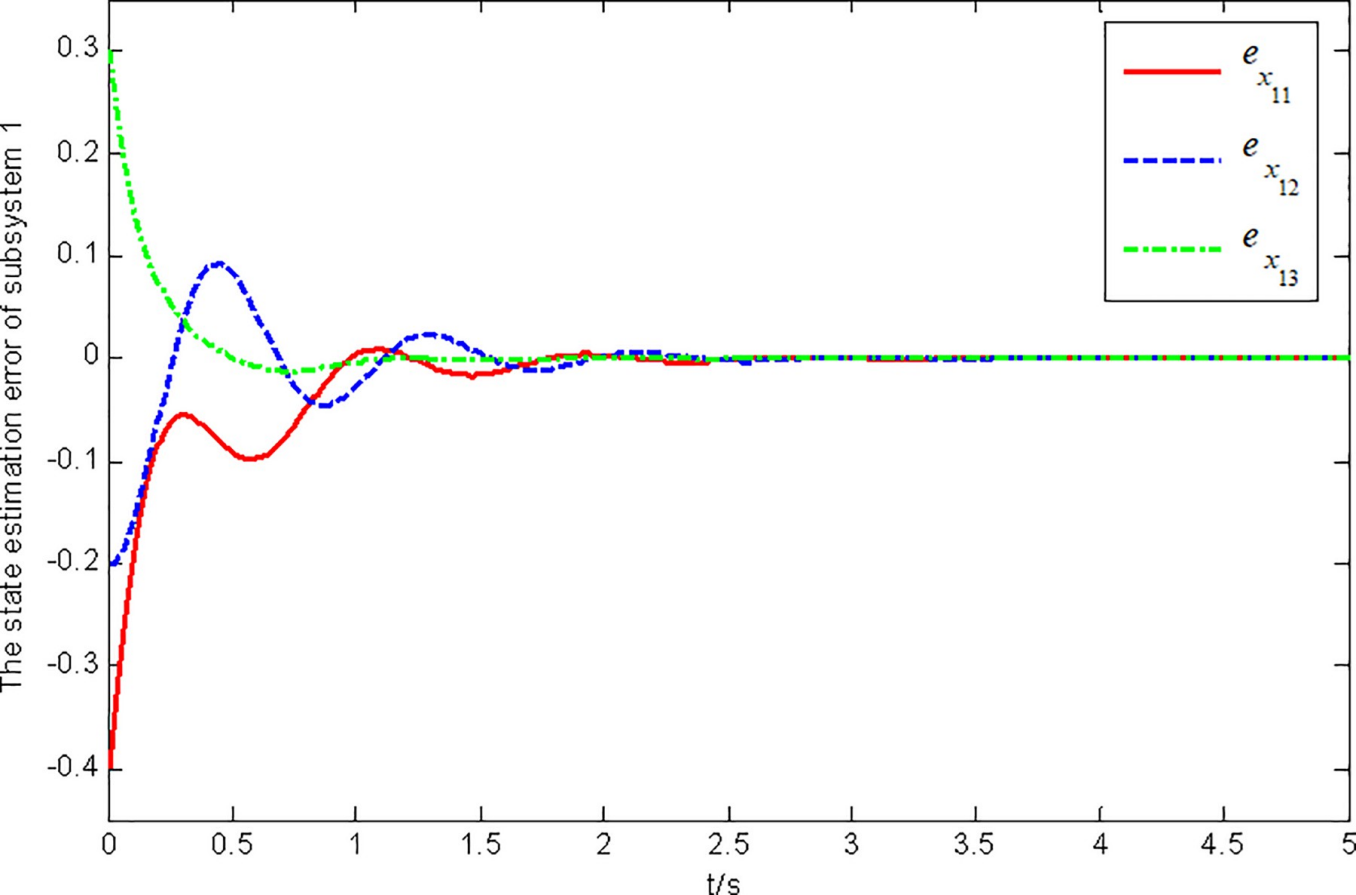
State estimation error response of subsystem 1 in interconnected system 2.

**Fig 6:**
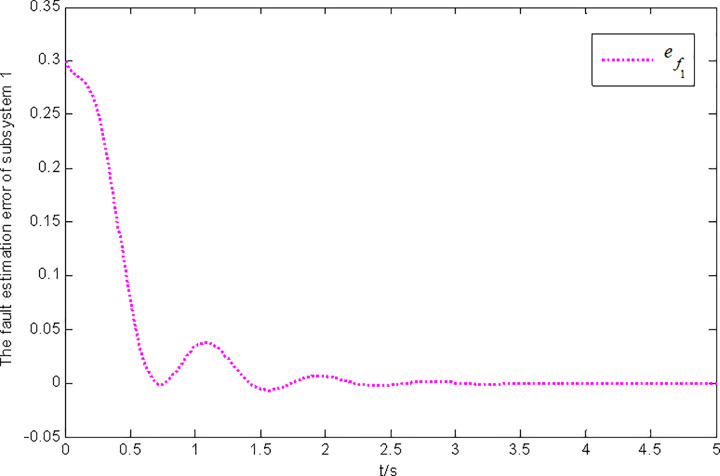
Fault estimation error response of subsystem 1 in interconnected system 2.

Figs 5 and 6 illustrate the state and fault estimation error response curves of subsystem 1 in interconnected system 2.

(2) Gain matrix of augmented observe in the first subsystem was assumed as:

$$L_{2}=(\begin{array}{cc} -0.3687 & 2.8911\\ -2.9450 & -0.3031\\ -0.4053 & -0.0456\\ -2.8702 & -0.5944 \end{array}).$$

**Fig 7:**
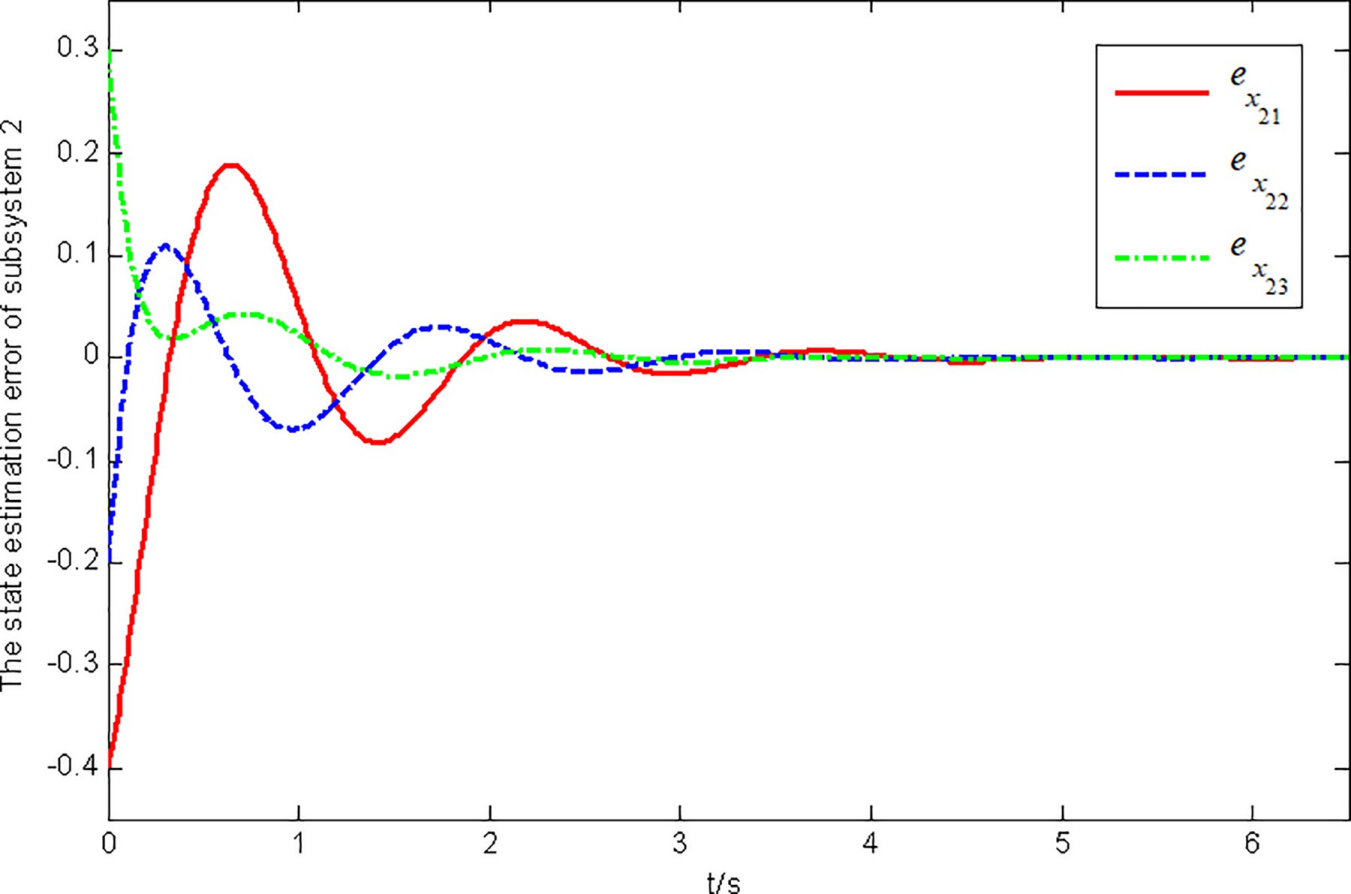
State estimation error response of subsystem 2 in interconnected system 2.

**Fig 8:**
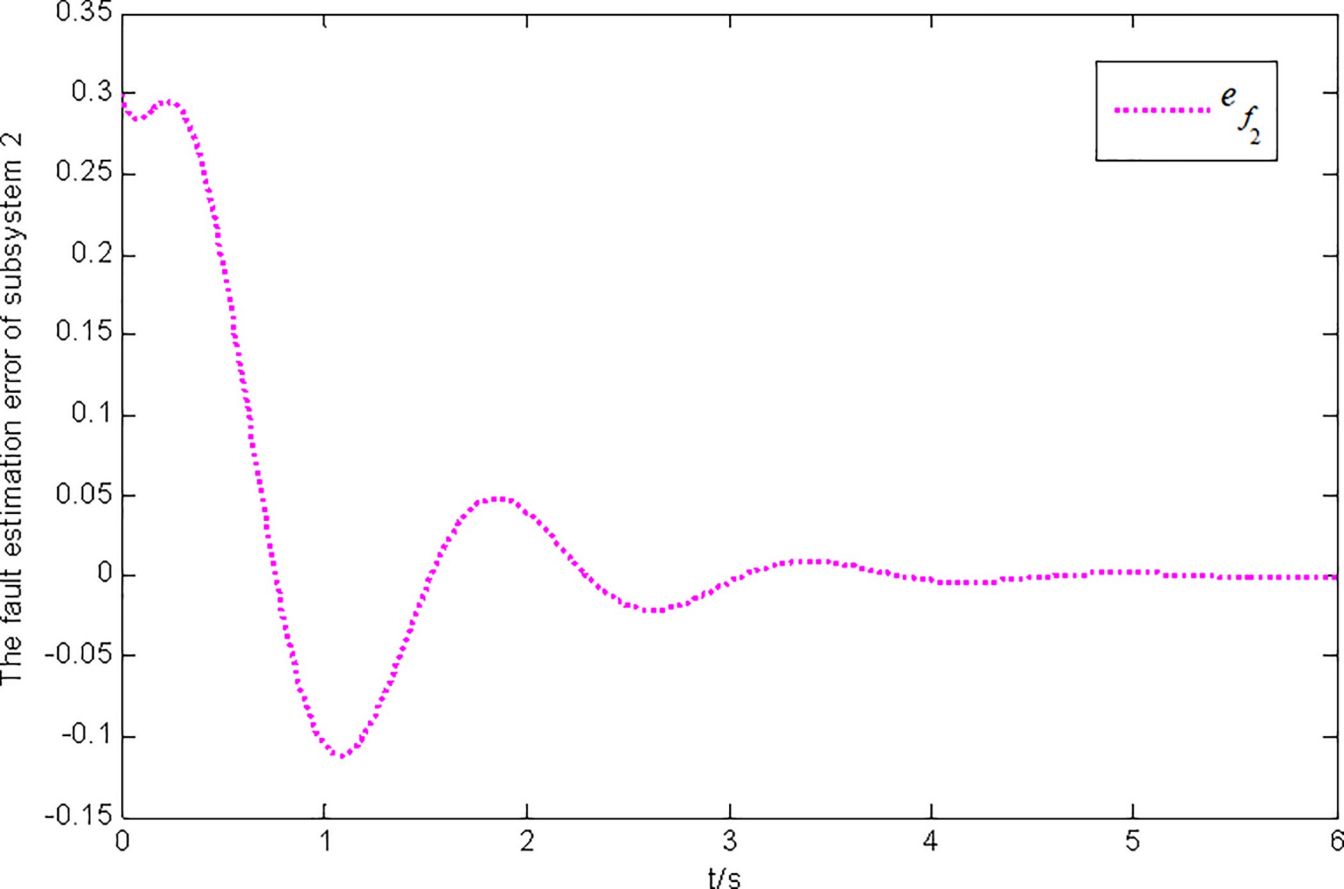
Fault estimation error response of subsystem 2 in interconnected system 2.

Figs 7 and 8 show the state and fault estimation error response curves of subsystem 2 in interconnected system 2.

From simulation results, it was seen that both state and fault estimation errors could converge to zero asymptotically. therefore, it was feasible and effective to utilize this method for robust estimation of sensor faults in interconnected systems.

**Remark 6.** In Illustrative examples, the gain matrix of the augmented observer can be solved by reference to the S1 File.

**Remark 7.** The example verified the correctness of the developed sensor fault estimation method of nonlinear interconnected systems based on the proposed observer and there was no specific requirement for system disturbance and fault parameters. Hence, the specific forms of disturbance and fault terms were not given.

## 6. Conclusions

In this paper, a generalized interconnected system is obtained by constructing augmentation vectors, and an augmented state fault observer is designed based on the generalized interconnected system. Considering the external disturbance in the system, performance indicators are set to reduce the influence of disturbance on fault estimation. Considering the efficiency of fault estimation, the error of fault estimation tends to zero rapidly by setting the index. At the same time, considering the convenience of observer design, the sufficient conditions for the existence of gain matrix are given in the form of matrix inequalities, which is more convenient for observer design. Finally, the proposed method is verified by two simulation examples, in which the state estimation error of two subsystems in interconnected system 1 can approach 0 within 2 seconds, and the fault estimation error needs to approach 0 within 6 seconds. The state estimation error of the two subsystems in interconnected system 2 tends to 0 after 4 seconds, and the fault estimation error tends to 0 after 5 seconds. It can be seen from Figs 2, 4, 6 and 8 that the proposed method can effectively estimate the sensor faults of each subsystem in the interconnected system within a limited time, which verifies the feasibility of the proposed fault estimation method. In future studies, the proposed method can provide reference for fault estimation methods of generalized and uncertain interconnected systems, and further enrich the fault estimation methods of complex large-scale systems.

## Supporting information

S1 File (PDF)
