# Supplementary material for: An estimation method for sensor faults based on observer in interconnected systems
Source: PLoS One. 2024 Mar 11;19(3):e0296848. doi: 10.1371/journal.pone.0296848 (PMC10927149; doi:10.1371/journal.pone.0296848)
Supplement: S1 File — (PDF) [file pone.0296848.s001.pdf]

The method for the gain matrix in this article

According to the linear matrix inequality matrix, the gain matrix can be solved:

According to the augmented matrix:

$$E = [I_n \quad 0], \bar{A}_i = [A_i \quad 0], \bar{C}_i = [C_i \quad G_i]$$

Solving matrix inequalities in papers.

5.1 Simulation verification of interconnected system without nonlinear term and disturbance term

a) Provide a constant matrix based on the paper;

b) Find S_i and T_i ;

C) Solving Matrix Inequalities:

```
setlmis([])
p1=lmivar(1,[3 1]);p2=lmivar(1,[3 1]);
y1=lmivar(2,[3 2]);y2=lmivar(2,[3 2]);
lmiterm([1 1 1 p1], a1'*t1',1); lmiterm([1 1 1 p1], 1,t1* a1); lmiterm([1 1 1 p1], 1,1);
lmiterm([1 2 2 p2], a2'*t2',1); lmiterm([1 2 2 p2], 1,t2* a2); lmiterm([1 2 2 p2], 1,1);
lmiterm([1 1 1 -y1], -c1',1); lmiterm([1 1 1 y1],-1, c1); lmiterm([1 2 2 -y2], -c2',1);
lmiterm([1 2 2 y2],-1, c2);
lmiterm([1 1 2 p1], 1,Te12);lmiterm([1 1 2 p2], Te21,1);
lmiterm([-2 1 1 p1],1,1);
lmiterm([-3 1 1 p2],1,1);
lmisys=getlmis;
[tmin,xfeas]=feasp(lmisys)
P1=dec2mat(lmisys,xfeas,p1);
P2=dec2mat(lmisys,xfeas,p2);
y1=dec2mat(lmisys,xfeas,y1);
y2=dec2mat(lmisys,xfeas,y2);
```

d) Solving the gain matrix:

```
l1=inv(p)*y1;
l2=inv(p)*y2.
```

5.2 Simulation verification of interconnected system with nonlinear and disturbance terms

a) Provide a constant matrix based on the paper;

b) Find S_i and T_i ;

C) Solving Matrix Inequalities:

```
setlmis([])
p1=lmivar(1,[4 1]);p2=lmivar(1,[4 1]);
y1=lmivar(2,[4 2]);y2=lmivar(2,[4 2]);
lmiterm([1 1 1 p1], 1,t1*a1,'s');lmiterm([1 4 4 p2], 1,t2*a2,'s'); lmiterm([1 1 1 p1], 1,1);
lmiterm([1 1 1 -y1], -c1',1); lmiterm([1 1 1 y1],-1, c1); lmiterm([1 4 4 -y2], -c2',1);
lmiterm([1 4 4 y2],-1, c2); lmiterm([1 4 4 p2], 1,1);
lmiterm([1 1 1 0],1.5);lmiterm([1 4 4 0],1.32);
lmiterm([1 1 2 p1], 1,1);lmiterm([1 4 5 p1], 1,1);
lmiterm([1 1 3 p1], 1,td1);lmiterm([1 4 6 p1], 1,td1);
lmiterm([1 2 2 0],-1);lmiterm([1 5 5 0],-1);
lmiterm([1 3 3 0],-1);lmiterm([1 6 6 0],-1);
lmiterm([1 1 4 p1], 1,Te12);lmiterm([1 1 4 p2], Te21,1);
lmiterm([-2 1 1 p1],1,1);
lmiterm([-3 1 1 p2],1,1);
lmisys=getlmis;
[tmin,xfeas]=feasp(lmisys)
P1=dec2mat(lmisys,xfeas,p1);
P2=dec2mat(lmisys,xfeas,p2);
y1=dec2mat(lmisys,xfeas,y1);
y2=dec2mat(lmisys,xfeas,y2);
```

d) Solving the gain matrix:

```
l1=inv(p)*y1;
l2=inv(p)*y2.
```
